# Real-life challenge: training program on drug use and adolescence in primary health care

**DOI:** 10.11606/s1518-8787.2019053001125

**Published:** 2019-08-12

**Authors:** Bruna Antunes de Aguiar Ximenes Pereira, Renata Cruz Soares de Azevedo

**Affiliations:** I Universidade Estadual de Campinas. Faculdade de Ciências Médicas. Programa de Pós-Graduação em Ciências Médicas. Campinas, SP, Brasil; II Universidade Estadual de Campinas. Faculdade de Ciências Médicas. Departamento de Psicologia Médica e Psiquiatria. Campinas, SP, Brasil

**Keywords:** Primary Health Care, human resources, Drug users, Triage, Disorders Related to Substance Use, prevention & control, Adolescent Health Services

## Abstract

Guidelines emphasize the importance of approaching substance use by adolescents, particularly in primary health care. However, there are problems with its incorporation. The objective of this study was to present the training stages on the theme for professionals in primary health care. Researchers conducted logistic structuring, content elaboration and evaluation of difficulties before and after training. Sixty percent of professionals involved in the care of adolescents in a medium-sized city participated in the study. More than half of them stated having difficulties in the approach, mainly theoretical limitations and short consultations. After the training, the professionals informed whether they felt more prepared, but practical difficulties remained.

## INTRODUCTION

Adolescence is the phase of greatest vulnerability to begin using psychoactive substances. Data from Brazil’s National Survey of School Health, conducted in 2015, indicated that rates of use of tobacco, alcoholic beverages and illicit psychoactive substances up to 15 years of age were 19%, 53% and 9%, respectively^1^.

The complexity of the factors involved in drug use in this age group makes it difficult to distinguish the adolescents who will maintain their use, with a chance of exposure to risks and evolution to dependence. Early identification and management can reduce deleterious consequences and professionals in primary health care stand out among the main actors for the establishment of these actions. The sensitization of managers, the motivation and the training of primary health care teams can contribute to modify this scenario, incorporating to the care routine triage tools, brief intervention and referral, if necessary. The main obstacles to this insertion are: lack of training, short consultation time, difficulty in leading positive cases, and pessimism regarding the screening benefits^2^.

The proposal of this study was to describe the training process of professionals in primary health care for identification and management of adolescents using psychoactive substances, the obstacles to their achievement and the difficulties faced by professionals before and after training.

## METHODS

This is a descriptive, qualitative and quantitative study, which presents the steps for training primary health care professionals to approach the use of psychoactive substances by adolescents. Eligible subjects were all health professionals in the city of Paulínia, state of São Paulo, who attended to adolescents in the city’s Basic Health Unit (UBS) during 2015 and 2016.

Initially, the Municipal Department was contacted and authorized participation in the training; the UBS were visited for presenting the proposal to the coordinators and teams. A training of 3 hours, with groups of 20 participants, in an easily accessible location was suggested.

At each meeting, sociodemographic data were anonymously collected: sex, age, profession, and time after graduation. For evaluating the professionals’ perception of success in the approach of adolescents using psychoactive substances, the close-ended question “Do you think that you are successful in approaching adolescents that are using alcohol or other drugs?” was applied before the training. For evaluating the difficulties of approaching drug use by adolescents in primary health care, the open-ended question “As a professional, what are the main difficulties of incorporating the approach to alcohol and other drug use among adolescents in your care routine at the UBS?” was applied before and after the training.

Exploratory analysis was held by summary measures of close-ended questions about sociodemographic data. To present the difficulties resulting from the open-ended question, before and after training, the researchers read the answers independently and grouped the difficulties mentioned by the professionals.

The research was approved by the Research Ethics Committee of the Faculdade de Ciências Médicas of the Universidade Estadual de Campinas (CAAE 41227514.6.0000.5404).

## RESULTS

We divided the steps for enabling the training process into four phases. In phase 1, we contacted the mental health technical advisor of the city to explain the project and schedule a meeting with the director of the Mental Health Department. Subsequently, we had a meeting together with the director of the Pediatrics Department and defined the professionals eligible for participation in the training, i.e. all those involved in the care of adolescents in primary health care. In phase 2, after authorization of the Health Department of city, we contacted the UBS to present the project, request the release of professionals for the training and for the delivery of the invitation letter. In phase 3, we conducted the first training cycle.

The content of the training included: physical and psychological characteristics of adolescence, brain development, national and international epidemiological data on substance use among adolescents, consumption patterns, importance of early identification, risk and protection factors, prevention in the health environment, Portuguese version^3^ of the instrument CRAFFT (acronym for Car; Relax; Alone; Forget; Family/Friends; Trouble) for screening and evaluation of seriousness. We also presented the Adolescent SBIRT algorithm^2^ (Screening, Brief Intervention and Referral for Treatment), as well as the guidelines for referrals within the city’s network, when necessary.

Aiming at an interactive activity, we produced digital material with the defined themes and made available a printed textbook for each participant, with more detailed content and space for complementary information and clarification of the doubts discussed.

Although we organized the training based on the feasibility explained by the managers, the training took us two training cycles, due to vacations, leaves of absences and difficulty to arrive at the course. Given this context, we added eight other dates (phase 4), in the morning and evening, contemplating weekdays not available in the previous training, in smaller groups, at the UBS.

Of the 126 primary care professionals attending to adolescents, 82 participated in the training. Of these, 59 were physicians, 21 were nurses and two were social workers, corresponding to 71%, 54% and 50% of professionals in each category, respectively, working in 2015 and 2016.

The mean age of participants was 43.9 years and the mean time after graduation was 19 years, with prevalence of female participants (77%).

In the survey before training, 64% professionals informed having difficulties in approaching psychoactive substance use by adolescents and 10% believed being successful in it (Table).

**Table:** Difficulties indicated before and after training regarding the approach to the use of psychoactive substances by adolescents in primary health care.

Pre-training	%
Confidentiality and presence of parents in consultation	24,4
Uncooperative posture of adolescent in consultation	20,7
Unprepared to approach the theme	20,7
Lack of adequate location to hold the approach	20,7
Short consultations and large volume of schedules	18,3
Not knowing what arguments to use to motivate the adolescent	13,4
Low frequency of adolescents in the basic health unit	12,2
Not knowing how to perform treatment and follow-up after approach and identification	10,9
Not having the habit of asking	9,7
Disliking this subject	9,7
Difficulty in maintaining the secrecy of information in consultations for relatives of employees of the basic health unit	9,7
Not knowing when and where to refer	9,7
Post-training	
Lack of experience to address the theme	30,5
Lack of time in the consultation	24,4
None (“I clarified my doubts,” “I feel safer”)	19,5
Difficulty in linking the adolescent to the basic health unit	14,6
Presence of parents in consultation	14,6
Incorporating into the daily care of consultation or into the routine consultations	14,6
Uncooperative posture of adolescent in consultation	13,4
Dealing with negligent family environment	13,4
Lack of adequate location to hold the approach	12,2
Lack of identification with subject	12,2
Volume of consultations	12,2
Lack of uniformity in conducting the work team	2,4

## DISCUSSION

The strategies for prevention of psychoactive substance use among adolescents, although recommended by reference bodies, remain little structured in primary health care^4^, making relevant the publicizing of training organization on the subject.

We prioritized availability of information and tools so that professionals would feel motivated and confident about the importance of their role in approaching the use of licit and illicit psychoactive substances in consultations. The objective was to stimulate professionals to insert the theme in the daily care and enable them to conduct triage and interventions based on the seriousness of each situation^2^.

There was an expressive participation of primary health care professionals of the studied city. For this, we adjusted the training logistics given the difficulties indicated by the managers. We suggest that future training programs privilege the participation of managers in the elaboration and execution of the training and adapt its structure to the needs of the context in which it will be applied.

The predominance of female professionals corroborates studies on primary health care. Values for mean age and time after graduation were higher than other studies^5^, possibly due to the career plan of this city, which favors the permanence of professionals.

Professionals highlighted lack of training and confidentiality as difficulties. Although the recommendation for the consultations with adolescents involves a moment alone with the health professional, we perceive that this practice needs to be reinforced and reassured in the training of primary health care teams. Especially in relation to the use of psychoactive substances, the approach is better executed without the presence of the guardian. Parents and adolescents should be informed about the confidentiality of the consultation, including limits for maintenance or breach of confidentiality^2^. As a suggestion, this topic should be particularly valued.

The professionals reported personal difficulties as limiting factor of their performance in the theme. It is known that the professionals’ beliefs about the use of psychoactive substances interfere in the capacity of intervention, generating more or less resistance on the part of the professional^4,5^. Studies indicate that training programs should privilege the demystification of conceptions that are not supported by the current scientific understanding on the theme and explore different dimensions on values and attitudes^4,5^.

The participants pointed out lack of time and volume of consultations as obstacles to approach adolescents in this context. This difficulty was noticed before and after training, even though the instrument and approach presented were brief. It is suggested that strategies such as application of the screening instrument before consultations or its self-application would optimize the professional’s time with each adolescent^2^. We should emphasize the importance of all professionals in adolescent health care, diluting the responsibilities and valuing different knowledge.

The low frequency of attendance and the adolescent’s posture were reported as factors that hindered the approach. However, the literature indicates that adolescents who use psychoactive substances are more likely to look for primary health care, and that 81% attended at least one consultation in primary health care service in the year prior to treatment^4^. Several reasons that motivate the entrance into the service can provide a door for the access and discussion of the use of psychoactive substances^2^. In addition, compared to formal services, primary health care offers quick and less stigmatizing access to interventions in adolescents for treatment of psychoactive substance use.

Difficulties about the approach itself should be central issues in training programs. It is extremely important to discuss the access to triage instruments of easy application, with good correlation with the clinic and the professional’s background to provide guidance on the risks related to the use of psychoactive substances in this age group, in an empathetic and comprehensive manner, opposed to confrontation strategies^2^.

Some professionals were not motivated to address the theme either before or after the training. Although the incorporation of the approach to psychoactive substance use into primary health care should be held by all professionals, teams can identify individuals with greater interest in its achievement, respecting individual differences^5^.

After the training, there was an increase in the self-perception of preparation to approach the theme, in accordance with the literature, which emphasizes that training plays an essential role in the motivation and preparation of professionals to question and intervene in this theme^4^. However, some difficulties remained after training, although less frequent. New obstacles emerged, such as incorporation of the approach to the routine and concern with the follow-up and family environment. We believe that these statements indicate reflection on the subject and healthy concerns on the learning process.

As a limitation in the data interpretation we emphasize the subjective character in grouping the answers about the difficulties mentioned and, although guaranteed anonymity, the possibility of inhibition on the information about difficulties after training.

Therefore, although there are indications of the invaluable relevance of primary health care professionals in the theme, it is necessary to provide tools so that they can play their role and contribute to modifying the current scenario. The availability of the training process, including its preparation and real-life difficulties, allow a frank discussion about its potentialities, limits and adjustments in face of the diversity of contexts in which primary health care professionals and adolescents are inserted, the main focus of our proposal.
